# Low prevalence of mobilized resistance genes *bla*_NDM_, *mcr-1*, and *tet*(X4) in *Escherichia coli* from a hospital in China

**DOI:** 10.3389/fmicb.2023.1181940

**Published:** 2023-05-19

**Authors:** Lin Sun, Guo-Zhuang Sun, Yue Jiang, Cai-Yue Mei, Zhen-Yu Wang, Han-Yun Wang, Gui-Mei Kong, Xinan Jiao, Jing Wang

**Affiliations:** ^1^Jiangsu Key Laboratory of Zoonosis/Jiangsu Co-innovation Center for Prevention and Control of Important Animal Infectious Diseases and Zoonoses, Yangzhou University, Yangzhou, China; ^2^Key Laboratory of Prevention and Control of Biological Hazard Factors (Animal Origin) for Agrifood Safety and Quality, Ministry of Agriculture of China, Yangzhou University, Yangzhou, China; ^3^Department of Clinical Laboratory, Xuyi People's Hospital, Huai'an, China; ^4^Medical School of Yangzhou University, Yangzhou University, Yangzhou, China

**Keywords:** carbapenem, colistin, tigecycline, patients, plasmid

## Abstract

The emergence and spread of carbapenemase genes, colistin resistance genes *mcr-1*, and tigecycline resistance gene *tet*(X) represent a significant threat to clinical therapy and public health. In this study, we investigated the presence of carbapenemase genes, *mcr-1*, and *tet*(X) in 298 *Escherichia coli* strains obtained from a teaching hospital in China. In total, eight (2.68%), six (2.01%), and one (0.34%) *E. coli* isolates carried *bla*_NDM_, *mcr-1*, and *tet*(X4), respectively. The *bla*_NDM_ gene was located on IncX3 (*n* = 4), F2:A-:B- (*n* = 3), and F2:A1:B1 (*n* = 1) plasmids, with high similarity to multiple plasmids belonging to the same incompatibility type from Enterobacteriaceae. Six MCR-producing strains contained *mcr-1*-carrying IncI2 plasmids, organized similarly to other *mcr-1*-bearing IncI2 plasmids from animals in China. The *bla*_CTX−M−55/64/132/199_ gene located within a typical transposition unit (IS*Ecp1*-*bla*_CTX−M_-*orf477*Δ) was inserted near *dnaJ* to generate 5-bp direct repeats in four *mcr-1*-positive plasmids. The *tet*(X) and another four resistance genes [*aadA2, tet*(A), *floR*, and Δ*lnu*(F)] were co-located on an IncX1 plasmid, highly similar to other *tet*(X4)-carrying IncX1 plasmids from *Escherichia* and *Klebsiella* of animal or food origin, except that the conjugative transfer region of IncX1 plasmids was absent in our plasmid. Although a low prevalence of *bla*_NDM_, *mcr-1*, and *tet*(X) was observed in *E. coli* from patients in this study, their dissemination associated with some successful pandemic plasmids is of great concern. The continued surveillance of these crucial resistance genes in patients should be strengthened.

## Introduction

Globally, multidrug-resistant (MDR) bacteria are rapidly increasing, which poses a serious threat to clinical therapy and public health (WHO, 2014). In 2019, six pathogens were responsible for 3.57 million deaths associated with antimicrobial resistance, with *Escherichia coli* as the leading pathogen (Antimicrobial Resistance Collaborators, 2022).

Carbapenems are the mainstream antimicrobial agents for treating severe infections caused by MDR Gram-negative pathogens (Wu et al., 2019). However, the emergence and global spread of carbapenem-resistant Enterobacteriaceae (CRE) have become a severe challenge in healthcare settings, particularly for New Delhi metallo-β-lactamase (NDM)-producing strains (Wu et al., 2019). *Escherichia coli* is one of the predominant carriers of *bla*_NDM_; some lineages, such as ST167, ST410, and ST617, and popular plasmids (IncX3, IncFII, and IncC) are associated with *bla*_NDM_ global dissemination in *E. coli* (Wu et al., 2019).

Colistin and tigecycline are used as last-resort antimicrobial agents for treating severe infections caused by MDR Gram-negative pathogens, especially CRE (Liu et al., 2016; Yaghoubi et al., 2022). However, the identification of novel plasmid-mediated colistin resistance gene *mcr-1* and tigecycline resistance gene *tet*(X4) in *E. coli* from pigs in China can reduce the clinical efficacy of these agents (Liu et al., 2016; He et al., 2019). Since the discovery of *mcr-1*, it has been reported in *E. coli* isolates from different sources worldwide, particularly from food animals. It has been frequently associated with epidemic plasmids (e.g., IncI2, IncX4, and IncHI2) (Nang et al., 2019). The *tet*(X4) gene has recently been increasingly reported in *E. coli* from different sources (e.g., animals, food, and humans), mainly in China (He et al., 2019; Fang et al., 2020). Mobile element IS*CR2* and various conjugative and mobilizable plasmids, such as IncQ, IncX1, IncFIB, and IncHI1, facilitate the horizontal transfer of *tet*(X4) in *E. coli* (Fang et al., 2020; Aminov, 2021).

A previous study reported the discovery of *mcr, tet*(X), and carbapenemases in human or animal gut microbiomes (Wang et al., 2020a). The spread of *mcr, tet*(X), and carbapenemases is worrisome, as options for therapy are limited. Therefore, there is an urgent need for evaluating and monitoring the prevalence of pathogenic bacteria carrying *mcr, tet*(X), and carbapenemases. In this study, we investigated the prevalence and characterization of mobilized resistance genes *tet*(X4), *mcr-1*, and *bla*_NDM_ in *E. coli* strains isolated from a teaching hospital in Jiangsu Province, China.

## Materials and methods

### Bacterial isolates and detection of *tet*(X), *mcr-1*, and carbapenemase genes

From November 2019 to November 2021, 298 non-duplicate *E. coli* strains were isolated from patients in a teaching hospital in Jiangsu Province, China. The presence of *tet*(X), *mcr-1*, and carbapenemase genes (*bla*_NDM_, *bla*_KPC_, *bla*_IMP_, *bla*_VIM_, and *bla*_OXA − 48_) was detected by PCR and sequencing according to previously described protocols (Poirel et al., 2011; Wang et al., 2018, 2019). The information on patients with *bla*_NDM_/*mcr-1*/*tet*(X)-carrying *E. coli* is described in Supplementary Table 1.

### Antimicrobial susceptibility testing

The *bla*_NDM_/*mcr-1*/*tet*(X)-carrying *E. coli* isolates were tested for susceptibility to 14 antimicrobial agents, namely, ampicillin, cefotaxime, meropenem, gentamycin, amikacin, streptomycin, tetracycline, tigecycline, chloramphenicol, nalidixic acid, ciprofloxacin, colistin, fosfomycin, and sulfamethoxazole/trimethoprim, by using the agar dilution method or broth microdilution method (limited to colistin and tigecycline). *E. coli* ATCC 25922 was used as the quality control strain. The results were interpreted according to Clinical and Laboratory Standards Institute (CLSI) M100, 30^th^ edition (CLSI, 2020). Streptomycin (>16 mg/L) was interpreted according to the epidemiological cutoff values for *E. coli* set by the European Committee on Antimicrobial Susceptibility Testing (EUCAST; www.eucast.org).

### Conjugation assay

Conjugation experiments were performed using *bla*_NDM_/*mcr-1*/*tet*(X)-carrying *E. coli* isolates as donor bacteria and *E. coli* C600 (high-level streptomycin resistance) as the recipient strain following a previously described protocol (Chen et al., 2007). In brief, the donor bacteria and *E. coli* C600 were grown in LB broth up to the logarithmic phase and then mixed in a 1:4 ratio and incubated at 37°C for 18 h. Transconjugants were selected on MacConkey or LB agar plates supplemented with 3,000 mg/L streptomycins and 2 mg/L of meropenem (for *bla*_NDM_), colistin (for *mcr-1*), or tigecycline [for *tet*(X)]. They were further confirmed by detecting the presence of *bla*_NDM_, *mcr-1*, and *tet*(X) using PCR. Transfer frequencies were calculated as the number of transconjugants per recipient. Conjugation experiments were performed at least in triplicate.

### Whole genome sequencing and analysis

Genomic DNA was extracted from all *bla*_NDM_/*mcr-1*/*tet*(X)-carrying *E. coli* isolates using a TIAN amp Bacteria DNA kit (Tiangen, Beijing, China) and sequenced by the Illumina Hiseq platform. The 150 bp pair-end raw reads were trimmed with the NGSQC toolkit v.2.3.3 and assembled into contigs using SPAdes v.3.8.2 (Bankevich et al., 2012). Three *E. coli* isolates, XYEH3314 (*bla*_NDM − 6_), XYEH3783 (*bla*_NDM − 5_), and XYEH3934 [*tet*(X4)], were sequenced using Nanopore MinION and assembled with Unicycler version 0.4.9. The remaining *bla*_NDM_/*mcr-1*-carrying plasmid contigs were assembled into a complete plasmid sequence using PCR and Sanger sequencing (Supplementary Table 2) using related plasmids as references by BLAST (http://blast.ncbi.nlm.nih.gov/Blast.cgi). The genome sequences were analyzed for multi-locus sequence typing (MLST) and resistance genes using MLST and ResFinder, respectively (https://cge.food.dtu.dk//services). Analysis and annotation of all *bla*_NDM_/*mcr-1*/*tet*(X4)-carrying plasmids were performed using the RAST server (Aziz et al., 2008), ISfinder (https://www-is.biotoul,fr/), ResFinder, PlasmidFinder (https://cge.food.dtu.dk/services/PlasmidFinder/), BLAST, and the Gene Construction Kit 4.5 (Textco BioSoftware, Inc., Raleigh, NC, United States). Blast Ring Image Generator (BRIG) version 0.95 was used to visualize the *bla*_NDM_-carrying plasmid comparisons (Alikhan et al., 2011).

### Phylogenetic analysis of *bla*_*NDM*_/*mcr-1*/*tet*(X4)-positive *Escherichia coli* strains

The phylogenetic tree of all *bla*_NDM_/*mcr-1*/*tet*(X4)-positive *E. coli* strains and *bla*_NDM_-positive *E. coli* ST167 in this study and the NCBI database was constructed based on core genome single nucleotide polymorphisms (SNPs) using ParSNP v. 1.2 (https://github.com/marbl/parsnp), respectively. ChiPlot (http://chiplot.online/#) was used to visualize the phylogenetic tree.

### Nucleotide sequence accession number

The genome sequences and *bla*_NDM_/*mcr-1*/*tet*(X4)-carrying plasmids have been deposited in GenBank under accession number PRJNA901528.

## Results and discussion

### Characterization of *bla*_*NDM*_-positive *E. coli* isolates

The *bla*_NDM_ gene has been globally distributed as the third most common carbapenemase-encoding gene (Wu et al., 2019). We detected eight (2.68%) *E. coli* isolates carrying the carbapenemase gene *bla*_NDM − 5_ (*n* = 7) or *bla*_NDM − 6_ (*n* = 1) (Table 1). None of the other tested carbapenemase genes were detected in this study. All NDM-producing *E. coli* were resistant to meropenem (MIC ≥ 8 mg/L), ampicillin, cefotaxime, gentamicin, tetracycline, chloramphenicol, nalidixic acid, ciprofloxacin, and sulfamethoxazole/trimethoprim (Table 1). Resistance to amikacin, streptomycin, or fosfomycin was observed in three, six, and four *bla*_NDM_-positive strains, respectively. Numerous resistance genes were identified in these *bla*_NDM_-carrying strains, which was consistent with their susceptibility profiles. Eight *bla*_NDM_-positive *E. coli* isolates were assigned to ST156 (*n* = 4), ST167 (*n* = 2), ST361 (*n* = 1), and ST1193 (*n* = 1), respectively (Table 1). *E. coli* ST167 has been a global clone for *bla*_NDM_ dissemination (Wu et al., 2019). ST156 has been frequently described as *bla*_NDM − 5_ carriers in patients and animals in China (Yang et al., 2016; Tang et al., 2019; Lin et al., 2020; Zhang et al., 2022). ST361 has been previously associated with *bla*_NDM − 5_ in patients worldwide (Park et al., 2020; Chakraborty et al., 2021; Huang et al., 2023). ST1193 has been the dominant ST of carbapenem-sensitive *E. coli* isolates in patients at an intensive care unit in China (Ding et al., 2023). Carbapenem-resistant *E. coli* ST1193, an isolate encoding NDM-5 found in this study, might be a cause for concern due to its ability to acquire carbapenem resistance and the potential to cause a future outbreak in hospitals. All of them could successfully transfer meropenem resistance and *bla*_NDM_ to *E. coli* C600 by conjugation at frequencies of 10^−6^ to 10^−5^ transconjugants per recipient (Supplementary Table 3).

**Table 1:** Characterization of *bla*_NDM_/*mcr-1*/*tet*(X4)-positive *E. coli* isolates in this study.

**Strain^a^**	**Sources^b^**	**Sampling Date**	**MLST**	**Resistance genes^c^**	**Resistance patterns^d^**	**Location^e^**	**Sequencing platform(s)**
XYEH933^*^	Urine, M	2020/6/18	ST167	*bla*_NDM − 5_/*bla*_TEM − 1b_/*bla*_CTX − M−55_/*aadA2/strAB/rmtB/tet*(A)*/floR/sul1/ sul2*/*dfrA12*	AMP/CTX/MEM/GEN/AMI/STR/TET/CHL/NAL/CIP/ SXT	F2:A-:B-	Illumina
XYEH944^*^	Urine, M	2020/6/18	ST167	*bla*_NDM − 5_/*bla*_TEM − 1b_/*bla*_CTX − M−55_*/aadA2/strAB/rmtB/tet*(A)*/floR/sul1/ sul2*/*dfrA12*	AMP/CTX/MEM/GEN/AMI/STR/TET/CHL/NAL/CIP/ SXT	F2:A-:B-	Illumina
XYEH1271	Blood, F	2020/8/6	ST156	*bla*_NDM − 5_/*bla*_TEM − 1b_*/aph(3')-Ia/aph(4)-Ia/aac(3)-Iva/tet*(B)*/floR/fosA3/sul2/dfrA12/mph*(A)	AMP/CTX/MEM/GEN/TET/CHL/NAL/CIP/FOS/SXT	IncX3	Illumina
XYEH1278	Blood, M	2020/8/6	ST156	*bla*_NDM − 5_/*bla*_TEM − 1b_*/aph(3')-Ia/aph(4)-Ia/aac(3)-Iva/tet*(B)*/floR/fosA3/sul2/dfrA12/mph*(A)	AMP/CTX/MEM/GEN/TET/CHL/NAL/CIP/ FOS/SXT	IncX3	Illumina
XYEH3314^*^	Urine, M	2021/6/25	ST361	*bla*_NDM − 6_*/bla*_TEM − 1b_/*bla*_CTX − M−55_*/aac(3)-IId/aadA1/aadA2/strAB/rmtB/tet*(A)*/floR/aac(6')-Ib-cr/qnrB6/sul1/sul2/dfrA12/dfrA27*/*mph*(A)/*erm*(B)/*arr-3*	AMP/CTX/MEM/GEN/AMI/STR/TET/CHL/NAL/CIP/ SXT	F2:A-:B-	Illumina, Nanopore
XYEH3520^*^	Urine, M	2021/7/19	ST156	*bla*_NDM − 5_/*bla*_TEM − 1b_/*bla*_CTX − M−55_/*bla*_CTX − M−65_*/aph(3')-Ia/aph(4)-Ia/aac(3)-Iva/aac(3)-IId/strAB/tet*(A)*/tet*(B)*/floR/fosA3/sul2/dfrA12/dfrA14/mph*(A)	AMP/CTX/MEM/GEN/STR/TET/CHL/NAL/CIP/FOS/ SXT	IncX3	Illumina
XYEH3521^*^	Urine, M	2021/7/19	ST156	*bla*_NDM − 5_/*bla*_TEM − 1b_/*bla*_CTX − M−55_/*bla*_CTX − M−65_*/aph(3')-Ia/aph(4)-Ia/aac(3)-Iva/aac(3)-IId/strAB/tet*(A)*/tet*(B)*/floR/fosA3/sul2/dfrA12/dfrA14/mph*(A)	AMP/CTX/MEM/GEN/STR/TET/CHL/NAL/CIP/FOS/ SXT	IncX3	Illumina
XYEH3783^*^	Urine, F	2021/8/31	ST1193	*bla* _NDM − 5_ */bla* _TEM − 1b_ / *aac(3)-IId/aadA1/aadA2/aadA22/tet(A)/floR/cml A1/qnrS1/sul3/dfrA12*	AMP/CTX/MEM/GEN/STR/TET/CHL/NAL/CIP/SXT	F2:A1:B1	Illumina, Nanopore
XYEH1135^*^	Urine, F	2020/7/21	ST4985	*mcr*−1/*bla*_CTX − M−132_/*bla*_OXA − 10_*/aph(3')-IIa/aadA1/tet*(A)*/floR/cmlA1/ dfrA14/arr-3*	AMP/CTX/GEN/STR/TET/CHL/NAL/CIP/CL/SXT	IncI2	Illumina
XYEH1313^*^	Urine, F	2020/8/12	ST450	*mcr-1/bla* _CTX − M−55_	AMP/CTX/NAL/CIP/CL	IncI2	Illumina
XYEH1459	Blood, F	2020/9/2	ST2003	*mcr-1/bla*_CTX − M−199_/*bla*_CTX − M−14_/*bla*_TEM − 1b_*/aac(3)-IId/aadA5/strAB/tet*(A)*/sul1/sul2/dfrA17/mph*(A)	AMP/CTX/GEN/STR/TET/NAL/CIP/CL/SXT	IncI2	Illumina
XYEH2259^*^	Urine, F	2021/1/13	ST88	*mcr*−1/*bla*_CTX − M−14_*/aph(3')-Ia/aph(4)-Ia/aac(3)-Iva/aadA1/aadA2/tet*(B)*/floR/cmlA1/oqxAB/ fosA3/sul1/sul2/sul3/dfrA12*	AMP/CTX/GEN/STR/TET/CHL/NAL/CIP/CL/FOS/SXT	IncI2	Illumina
XYEH2299^*^	Urine, F	2021/1/20	ST88	*mcr*−1/*bla*_CTX − M−14_*/aph(3')-Ia/aph(4)-Ia/aac(3)-Iva/aadA1/tet*(B)*/floR/cmlA1/oqxAB/ fosA3/sul1/sul2/sul3/dfrA12*	AMP/CTX/GEN/STR/TET/CHL/NAL/CIP/CL/FOS/SXT	IncI2	Illumina
XYEH3022	Urine, M	2021/5/16	ST617	*mcr-1/bla*_CTX − M−64_/*bla*_TEM − 1b_*/aph(3')-Ia/aph(4)-Ia/aac(3)-Iva/aadA2/strAB/rmtB/tet*(A)*/floR/ fosA3/sul1/sul2/dfrA12/erm*(B)*/mph*(A)	AMP/CTX/GEN/AMI/STR/TET/CHL/NAL/CIP/CL/ FOS/SXT	IncI2	Illumina
XYEH3934	Urine, F	2021/9/19	ST7131	*bla*_CTX − M−14_*/aadA1/aadA2/strAB/*tet*(A)/*tet*(X4)*/floR*/cmlA1/qnrS1/ fosA3/sul3/dfrA12/dfrA14/mef*(B)*/*lnu*(F)*	AMP/CTX/STR/TET/TIL/CHL/NAL/FOS/SXT	IncX1	Illumina, Nanopore

^*a*, *^indicates that the strain could successfully transfer bla_*NDM*_*/mcr-1 to E. coli C600 by conjugation*. ^*b*^*F, female; M, male*. ^*c*^*Resistance genes located with bla*_*NDM*_*, mcr-1, or tet(X4) on the same plasmid are underlined. Antibiotic resistance genes with* >*90% sequence homology and coverage are shown*. ^*d*^*AMP, ampicillin; CTX, cefotaxime; MEM, meropenem; GEN, gentamycin; AMI, amikacin; STR, streptomycin; TET, tetracycline; TIL, tigecycline; CHL, chloramphenicol; NAL, nalidixic acid; CIP, ciprofloxacin; CL, colistin; FOS, fosfomycin; SXT, sulfamethoxazole/trimethoprim*. ^*e*^*Plasmids carried bla*_*NDM*_*/mcr-1/tet(X4)*.

**Figure 1:**
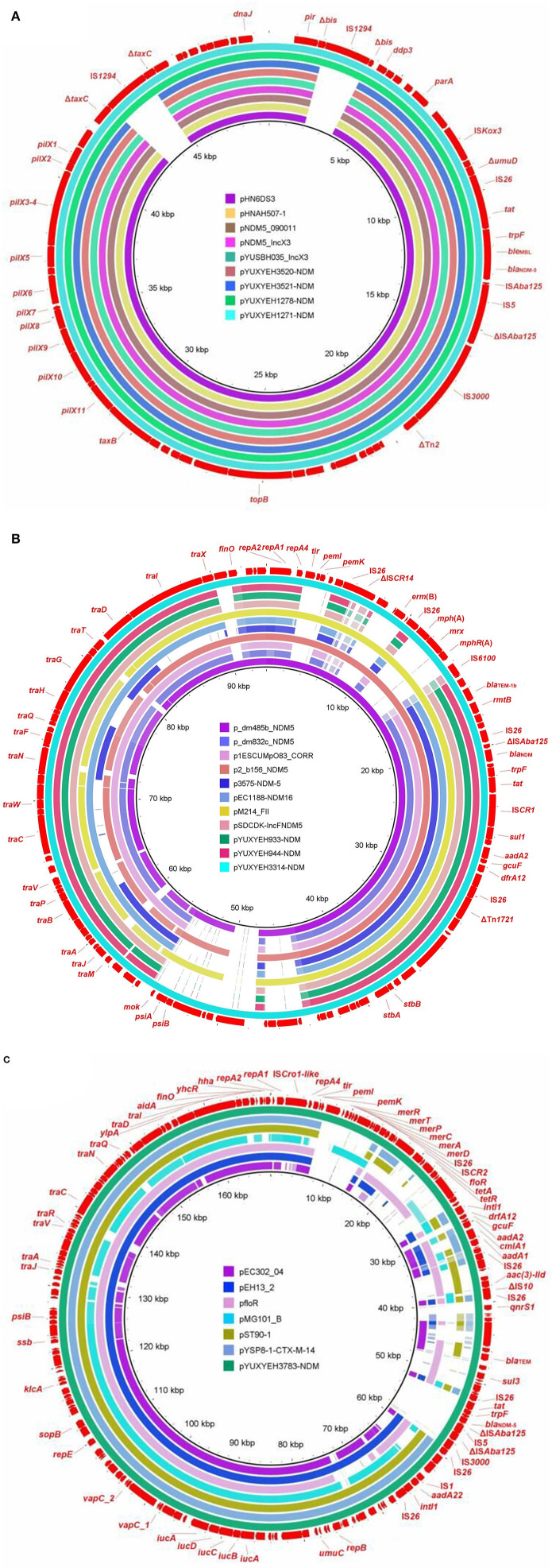
Sequence comparison of **(A)** IncX3 plasmids pYUXYEH1271-NDM, pYUXYEH1278-NDM, pYUXYEH3520-NDM, and pYUXYEH3521-NDM in this study with other NDM-producing plasmids pHN6DS3 (MN276078, *Enterobacter cloacae*, dog, China), pHNAH507-1 (MH286945, *Escherichia coli*, chicken feces, China), pNDM5_090011 (CP036312, *E. hormaechei*, human, China), pNDM5_IncX3 (KU761328, *Klebsiella pneumoniae*, human, China), and pYUSBH035_IncX3 (CP099447, *K. quasipneumoniae*, human, China) **(B)** F2:A-:B- plasmids pYUXYEH933-NDM, pYUXYEH944-NDM, and pYUXYEH3314-NDM in this study with other NDM-producing plasmids p_dm485b_NDM5 (CP095622, *K. pneumoniae*, human, Bangladesh), p_dm832b_NDM5 (CP095656, *E. coli*, human, Bangladesh), p1ESCUMpO83_CORR (CP034254, *E. coli*, mastitis milk, India), p2_b156_NDM5 (CP095683, *K. pneumoniae*, human, Bangladesh), p3575-NDM-5 (CP048012, *E. coli*, human, Spain), pEC1188-NDM16 (MH213345, *E. coli*, human, China), pM214_FII (AP018144, *E. coli*, human, Myanmar), and pSDCDK-IncFNDM5 (MT621569, *E. coli*, chicken farm, China) **(C)** F2:A1:B1 plasmid pYUXYEH3783-NDM in this study with other F2:A1:B1 plasmids pEC302_04 (CP011493, *E. coli*, human, Malaysia), pMG101_B (CP070963, *E. coli*, USA), pYSP8-1-CTX-M-14 (CP037912, *E. coli*, pig, China), or F2:A1:B- plasmids pfloR (CP047011, *E. coli*, pig, China), pST90-1 (CP050735, *Salmonella* Typhimurium, duck, China), and pEH13_2 (CP089099, *K. pneumoniae*, hospital, Hong Kong). The outer circle in red with annotation is the reference plasmid pYUXYEH1271-NDM, pYUXYEH3314-NDM, or pYUXYEH3783-NDM obtained in this study.

**Figure 2:**
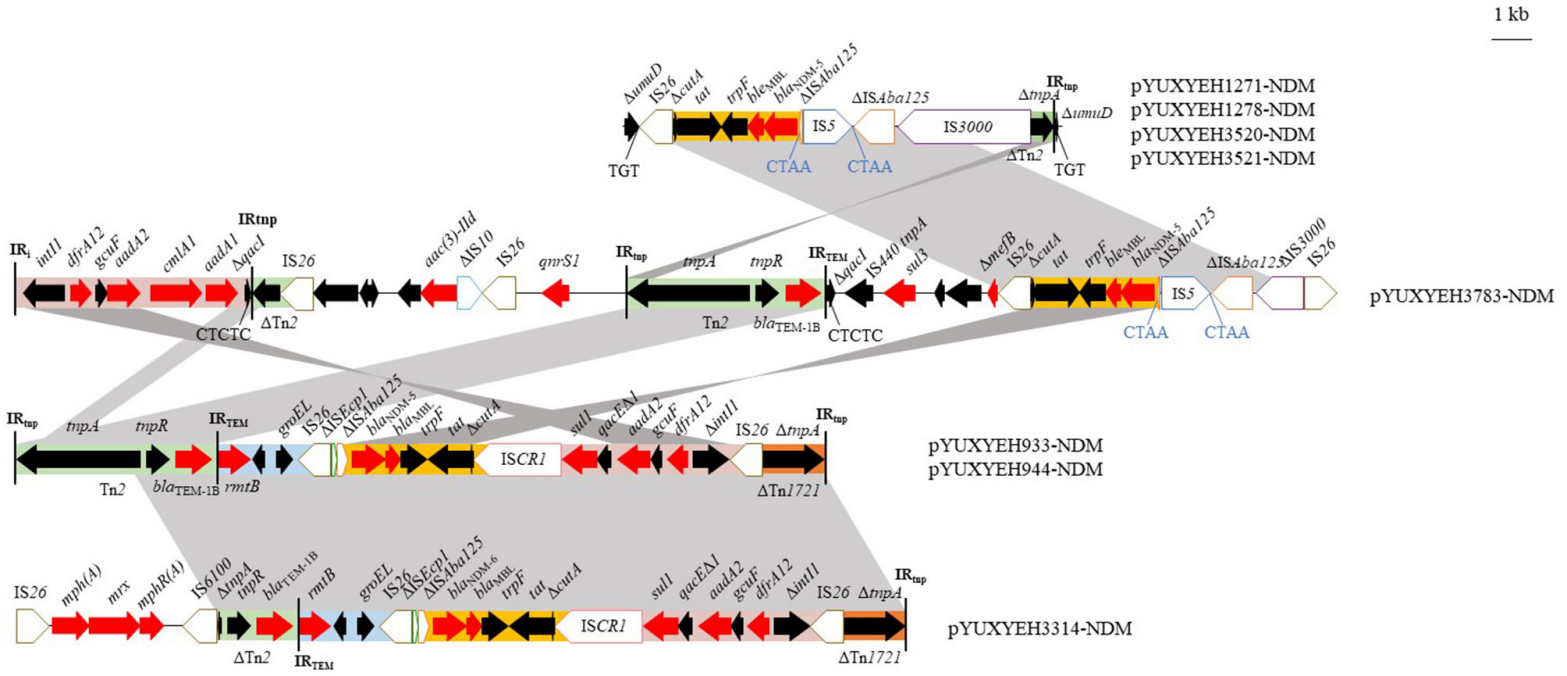
Genetic structures of *bla*_NDM_ in plasmids pYUXYEH933-NDM, pYUXYEH944-NDM, pYUXYEH1271-NDM, pYUXYEH1278-NDM, pYUXYEH3314-NDM, pYUXYEH3520-NDM, pYUXYEH3521-NDM, and pYUXYEH3783-NDM obtained in this study. The extent and directions of genes are indicated by arrows. Δ indicates a truncated gene or mobile element. Insertion sequences (ISs) are shown as boxes labeled with their names. Direct repeats are indicated by arrows and sequences. Tall bars represent the inverted repeats (IRs) of a transposon or integron. Regions with >99% identity are shaded in gray.

The *bla*_NDM_ gene was located on IncX3 (*n* = 4), F2:A-:B- (*n* = 3), and F2:A1:B1 (*n* = 1) plasmids (Table 1). The IncX3 plasmids carried only one resistance gene, *bla*_NDM − 5_, but the IncFII plasmids also carried some other resistance genes, such as *bla*_TEM − 1b_, *aadA2*, and *dfrA12* (Table 1). Four *bla*_NDM − 5_-carrying IncX3 plasmids (pYUXEEH1271-NDM, pYUXEEH1278-NDM, pYUXYEH3520-NDM, and pYUXYEH3521-NDM) were obtained from four ST156 NDM-5-producing *E. coli* strains. These IncX3 plasmids were identical, except that two copies of IS*1294* were inserted into the accessory replication gene *bis* and DNA relaxase gene *taxC*, respectively, in pYUXEEH1271-NDM and pYUXEEH1278-NDM (Figure 1A). They were also identical or highly similar to other NDM-producing IncX3 plasmids from Enterobacteriaceae (e.g., *E. coli, Enterobacter*, and *Klebsiella*) of animal or human origin (Figure 1A). Two F2:A-:B- plasmids, pYUXYEH933-NDM (82,619 bp) and pYUXYEH944-NDM (82,628 bp), obtained from two ST167 NDM-5-encoding isolates, were highly similar and differed by only one nucleotide and one more 9-bp tandem repeat (CAACAGCCG) in the *traD* gene in pYUXYEH944-NDM (Figure 1B). The *bla*_NDM_ gene in isolates XYEH3314 and XYEH3783 was located on F2:A-:B- plasmid pYUXYEH3314-NDM (*bla*_NDM − 6_, 93,647 bp) and F2:A1:B1 plasmid pYUXYEH3783-NDM (*bla*_NDM − 5_, 168,068 bp). These IncFII plasmids showed high similarity to multiple plasmids belonging to the same incompatibility type from Enterobacteriaceae (e.g., *E. coli, K. pneumoniae*, and *Salmonella*) from food-producing animals or humans (Figures 1B, C). These *bla*_NDM_-carrying plasmids shared the same core structure Δ*cutA*-*tat*-*trpF*-*ble*_MBL_-*bla*_NDM − 5/−6_-ΔIS*Aba125* (Figure 2). IncX3 and IncFII plasmids are major vectors for the global transmission of *bla*_NDM_ in *Enterobacteriaceae* (Wu et al., 2019). Particularly, *bla*_NDM_-carrying IncX3 plasmids have been widely disseminated among *E. coli* strains from patients, food-producing animals, companion animals, food products, and the environment in China (Yang et al., 2016; Wu et al., 2019; Zhang et al., 2019; Cen et al., 2021; Wang et al., 2021; Zhao et al., 2021).

### Characterization of *mcr-1*-carrying *E. coli* isolates

To date, 10 plasmid-mediated *mcr* variants (*mcr-1* to *mcr-10*) have been identified in *Enterobacteriaceae* from animals, humans, and the environment (Hussein et al., 2021). Among them, *mcr-1* is the most common *mcr* variant worldwide (Hussein et al., 2021). In this study, six (2.01%) *E. coli* strains were positive for the colistin resistance gene *mcr-1*. The prevalence of *mcr-1* is close to 4.2%, which is low, in human clinical isolates worldwide (Bastidas-Caldes et al., 2022). The prevalence of *mcr-1* in *E. coli* was significantly decreased in both animals and humans in China after banning colistin as a growth promoter on 30 April 2017 (Wang et al., 2020b).

All *MCR-1*-positive strains were resistant to colistin (MIC = 8 mg/L), ampicillin, cefotaxime, nalidixic acid, ciprofloxacin, and some other agents (Table 1). In addition to *mcr-1*, they also carried other clinically important resistance genes, such as *bla*_CTX − M_ (*n* = 6), *tet*(A) (*n* = 3), *fosA3* (*n* = 3), and *rmtB* (*n* = 1) (Table 1). The presence of these resistance genes could explain their multi-resistance profiles. Six MCR-1-producing isolates were assigned to five STs (ST88, ST450, ST617, ST2003, and ST4985) (Table 1).

**Figure 3:**
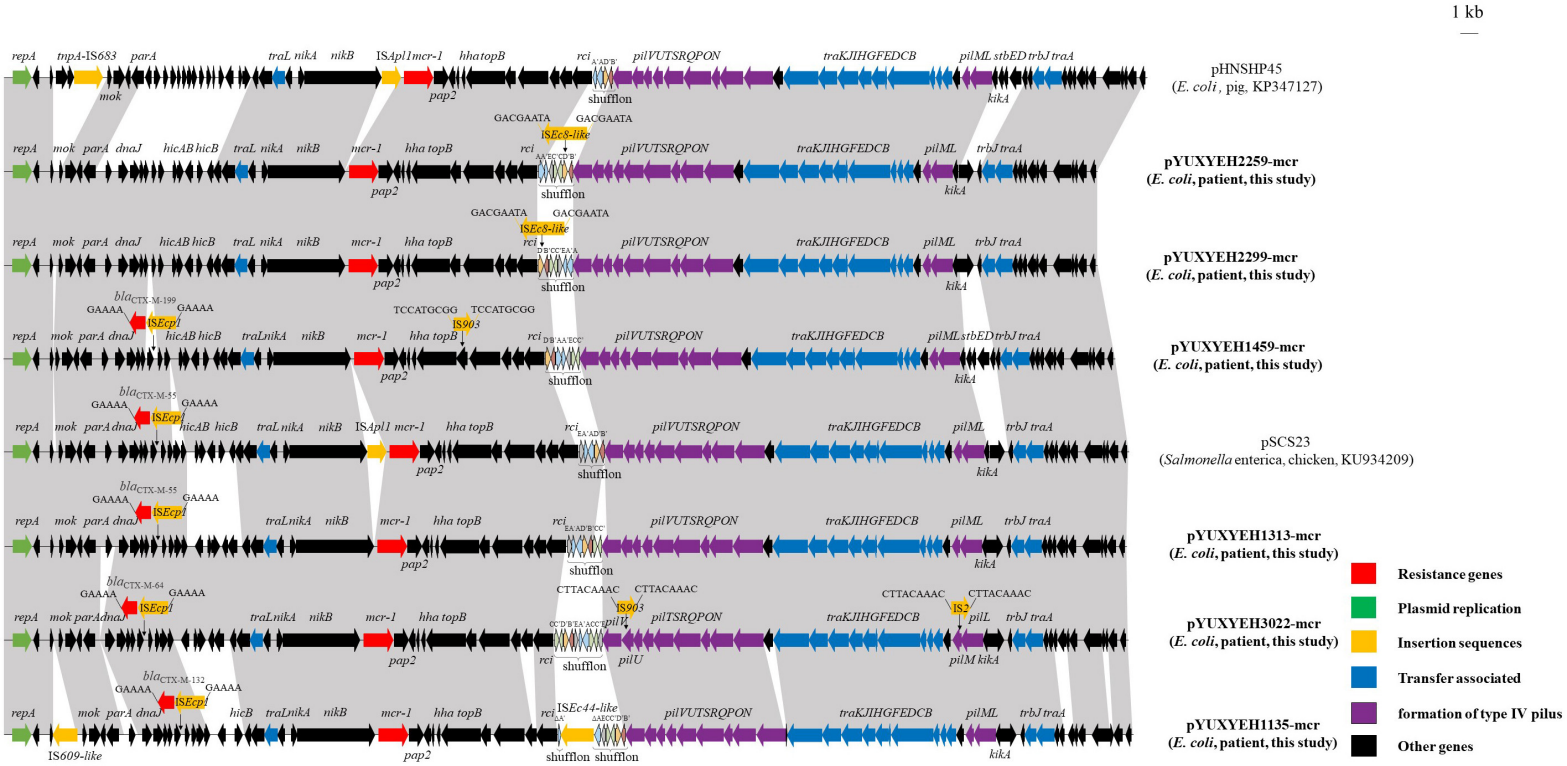
Linear comparisons of plasmids pYUXYEH1135-mcr, pYUXYEH1313-mcr, pYUXYEH1459-mcr, pYUXYEH2259-mcr, pYUXYEH2299-mcr, and pYUXYEH3022-mcr in this study with other IncI2 *mcr-1*-carrying plasmids. The extent and directions of genes are indicated by arrows. Labeled vertical arrows with an IS box indicate the insertion site of the IS element. Direct repeats are indicated by arrows and sequences. Regions with >99% identity are shaded in gray.

Among them, four *mcr-1*-positive *E. coli* strains could transfer *mcr-1* to *E. coli* C600 by conjugation with frequencies of 10^−6^ to 10^−4^ transconjugants/recipient (Supplementary Table 3). The *mcr-1* gene was located on IncI2 plasmids in all six strains. These *mcr-1*-carrying plasmids with sizes between 63,409 and 67,529 bp were organized similarly to other *mcr-1*-bearing IncI2 plasmids from food-producing animals in China, such as the first identified *mcr-1*-positive plasmid pHNSHP45 (pig, KP347127) from *E. coli* and pSCS23 (chicken, KU934209) from *Salmonella* (Figure 3). However, IS*Apl1*, located upstream of *mcr-1* in pHNSHP45 and pSCS23, was absent in our plasmids (Figure 3). The rapid global dissemination of *mcr-1* is largely attributed to horizontal transfer via some successful plasmids such as IncX4, IncI2, and IncHI2 (Bastidas-Caldes et al., 2022).

Notably, four *mcr-1*-bearing plasmids in this study also carried the extended-spectrum β-lactamase gene *bla*_CTX − M_ (*bla*_CTX − M−55/64/132/199_) (Table 1). The *bla*_CTX − M_ gene, located within a typical transposition unit (IS*Ecp1*-*bla*_CTX − M_-*orf477*Δ), was inserted near *dnaJ* to generate 5-bp direct repeats (5′-GAAAA-3′) (Figure 3). The co-location of *bla*_CTX − M_ would allow for *mcr-1* co-selection under cephalosporins, which have been widely used to treat clinical infections.

### Characterization of a *tet*(X4)-positive *E. coli* isolate

Since the discovery of the plasmid-mediated tigecycline resistance gene *tet*(X4) in *E. coli* from pigs in China in 2019 (He et al., 2019), it has been identified in *E. coli* from food-producing animals, wild birds, food products, and the environment (Chen et al., 2019; Li et al., 2021; Mohsin et al., 2021). Although tigecycline is applied in a limited way in humans, *tet*(X4) has been rarely reported in *E. coli* from patients. It was detected in 4.5% (45/1,001) fecal samples of hospital patients in Guangdong province, and only 0.07% (3/4,212) *E. coli* isolates from patients in China were identified to carry *tet*(X4) (He et al., 2019; Cui et al., 2022). We detected only one (0.34%) *tet*(X4)-positive *E. coli* isolate, XYEH3934. This strain exhibited high-level tigecycline resistance (MIC = 32 mg/L) and was also resistant to ampicillin, cefotaxime, streptomycin, tetracycline, chloramphenicol, nalidixic acid, fosfomycin, and sulfamethoxazole/trimethoprim (Table 1). However, it failed to transfer tigecycline resistance and *tet*(X4) to *E. coli* C600 by conjugation.

**Figure 4:**
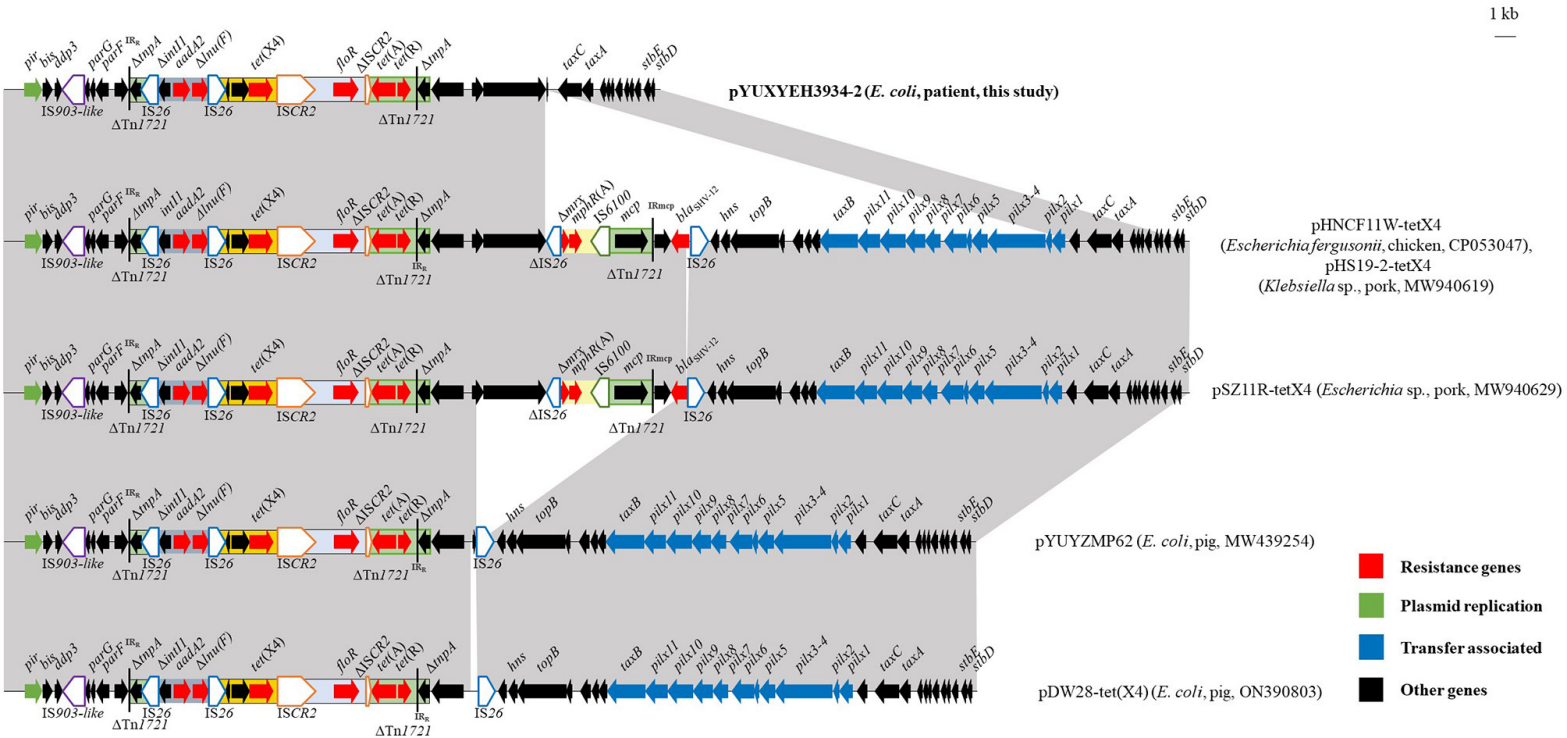
Linear comparisons of plasmid pYUXYEH3934-2 in this study with other IncX1 *tet*(X4)-carrying plasmids. The extent and directions of genes are indicated by arrows. Δ indicates a truncated gene or mobile element. Insertion sequences (ISs) are shown as boxes labeled with their names. Tall bars represent the 38-bp inverted repeats (IRs) of the transposon. Regions with >99% identity are shaded in gray.

The complete sequence of isolate XYEH3934 was obtained. It comprised one chromosome (4,709,641 bp) and four plasmids (Supplementary Table 4). Among them, *tet*(X) and another four resistance genes [*aadA2, tet*(A), *floR*, and Δ*lnu*(F)] were co-located on an IncX1 plasmid pYUXYEH3934-2 with a size of 31,816 bp. It was highly similar to other *tet*(X4)-carrying IncX1 plasmids from porcine *E. coli* in China, such as plasmids pYUYZMP62 (MW439254) and pDW28-tet(X4) (ON390803), as well as *tet*(X4)-positive plasmids from *Escherichia* and *Klebsiella* (Figure 4). However, the conjugative transfer region of IncX1 plasmids was absent in pYUXYEH3934-2 (Figure 4), which may explain the failure of conjugation. As previously described (Liu et al., 2022), *tet*(X4) was also associated with IS*CR2* in pYUXYEH3934-2 (Figure 4). The rapid dissemination of *tet*(X4) is mainly mediated by the horizontal transfer of insertion sequences (e.g., IS*CR2* and IS*1*) and plasmids such as IncX1 in the present study (Aminov, 2021; Liu et al., 2022; Yu et al., 2022). However, the clonal spread of *tet*(X4)-positive *E. coli* strains has been previously described, such as *E. coli* ST877, ST10, and ST48 between animals and humans (Cui et al., 2022) and *E. coli* ST761 among different sources (Wang et al., 2022; Zhai et al., 2022).

### Phylogenomic analysis of *bla*_*NDM*_/*mcr-1*/*tet*(X4)-positive *E. coli* isolates

We performed a phylogenomic analysis based on cgSNP to compare the genetic differences between *bla*_NDM_/*mcr-1*/*tet*(X4)-positive *E. coli* isolates in the present study. The results showed that the 15 *E. coli* strains were divided into four clades (Supplementary Figure 1). Some *E. coli* strains with the same STs exhibited close genetic relationships with identical or close antimicrobial susceptibility profiles and resistance genes, such as two *bla*_NDM − 5_-carrying ST167 *E. coli* isolates, two *mcr-1*-carrying ST88 *E. coli* isolates, and four *bla*_NDM − 5_-carrying ST156 *E. coli* isolates (Supplementary Figure 1). It suggests that clonal spread may occur in this teaching hospital in Jiangsu province. Since *E. coli* ST167 has been a global epidemic clone for *bla*_NDM_ dissemination, we further compared the genetic relationship between NDM-5-producing ST167 *E. coli* isolates in the present study and 33 ST167 *E. coli* from the NCBI database. Two NDM-encoding *E. coli* isolates, XYEH933 and XYEH944, and 13 NDM-5-producing ST167 *E. coli* from China (*n* = 12) and Italy (*n* = 1) were clustered in one clade with a close relationship (Supplementary Figure 2).

## Conclusions

Although a low prevalence of *bla*_NDM_, *mcr-1*, and *tet*(X) was observed in *E. coli* isolates from patients in one hospital in this study, their dissemination in *E. coli* associated with some successful pandemic plasmids (IncX, IncF, and IncI2) is of great concern. Conversely, *E. coli* strains from food or food-producing animals in China show a higher prevalence of *bla*_NDM_, *mcr-1*, and *tet*(X) (He et al., 2019; Zhang et al., 2019; Zhao et al., 2020; Cen et al., 2021). However, the small number of *E. coli* strains detected is a limitation of this study. Plasmids facilitate the rapid and global dissemination of resistance genes in Enterobacteriaceae from various sources. Given that resistant bacteria or plasmids could be transmitted from animals to humans via the food chain, the environment, or close contact, surveillance and control of these medically important resistance genes in patients are warranted and need to be optimized based on the One Health approach.

## Data availability statement

The datasets presented in this study can be found in online repositories. The names of the repository/repositories and accession number(s) can be found below: https://www.ncbi.nlm.nih.gov/, PRJNA901528.

## Author contributions

LS and JW conceived the study. LS, G-ZS, YJ, C-YM, H-YW, and G-MK carried out the experiments. LS, YJ, Z-YW, and JW analyzed the data. JW wrote the manuscript. XJ revised the manuscript. All authors have read and approved the final version of the manuscript.
