# Supplementary material for: Low prevalence of mobilized resistance genes blaNDM, mcr-1, and tet(X4) in Escherichia coli from a hospital in China
Source: Front Microbiol. 2023 May 19;14:1181940. doi: 10.3389/fmicb.2023.1181940 (PMC10237293; doi:10.3389/fmicb.2023.1181940)
Supplement: Supplementary file 1 [file Data_Sheet_1.PDF]

Table S1 Information of patients harbouring *bla*$_{NDM}$/*mcr-1*/*tet*(X4)-positive *Escherichia coli*

Isolate	illness	Antibiotic therapy	Outcomes
XYEH933	malignant tumor	ceftazidime	Treatment abandoning
XYEH944	malignant tumor	ceftazidime	Treatment abandoning
XYEH1271	diabetes	amoxicillin/clavulanate, amikacin	no abnormalities, discharge
XYEH1278	diabetes	amoxicillin/clavulanate, amikacin	no abnormalities, discharge
XYEH3314	malignant tumor	ceftizoxime	no abnormalities, discharge
XYEH3520	urinary tract infection	ceftazidime, amikacin	cured
XYEH3521	urinary tract infection	ceftazidime, amikacin	cured
XYEH3783	urinary tract infection	ceftizoxime	cured
XYEH1135	urinary tract infection	ceftazidime	cured
XYEH1313	diabetes	amoxicillin/clavulanate, ofloxacin	cured
XYEH1459	lupus erythematosus	-	Transfer to another hospital
XYEH2259	fever	ceftizoxime, sulperazone, moxifloxacin	cured
XYEH2299	postoperative urinary tract infection	cefazolin, ofloxacin	cured
XYEH3022	urinary tract infection	ceftizoxime	cured
XYEH3934	abdominal pain	cefuroxime	cured

-: do not use antibiotics

Table S2 Primers used to assemble *bla*$_{\text{NDM}}$/*mcr-1*-carrying plasmids

Region	sequence (5'-3')	Size (bp)	Position (bp) in plasmid
hp-IS*609*	F:ATCACTTGCGGAATAA	1742	2495-2510 (pYUXYEH1135-mcr)
	R:GTAAAGGGCGGTAAA		4222-4236 (pYUXYEH1135-mcr)
IS*Ecp1-hp*	F:AGCCACTATCCATTC	1483	11636-11650 (pYUXYEH1135-mcr)
	R:ACTGTTGTCCCGTAT		13104-13118 (pYUXYEH1135-mcr)
Δ*orf477-bla*$_{\text{CTX-M}}$	F:CGTAGGAGCATACTGAT	1524	8054-8070 (pYUXYEH1313-mcr)<br>7872-7888 (pYUXYEH1459-mcr)
	R:ATTGAAAGGTGGTTGT		9562-9577 (pYUXYEH1313-mcr)<br>9377-9392 (pYUXYEH1459-mcr)
bla$_{\text{CTX-M}}$-IS*Ecp1*	F:GCATACAGCGGCACA	1940	9342-9356 (pYUXYEH1313-mcr)<br>9160-9174 (pYUXYEH1459-mcr)
	R:CTACGGGCGCAACAA		11267-11281 (pYUXYEH1313-mcr)<br>11082-11096 (pYUXYEH1459-mcr)
IS*Ec8-like-pilV*	F:TCAGAGCCGAAGAACA	2762	31586-31601 (pYUXYEH2259-mcr)
	R:CAGCGTGAATAATAAAGG		34330-34347 (pYUXYEH2259-mcr)
parA-hp	F:TTCGTTCCAGCTAAG	978	4914-4928 (pYUXYEH2299-mcr)
	R:ATAATCACGCACTCG		5877-5891 (pYUXYEH2299-mcr)
IS*Ec8-like*	F: TATGACGCCAAGCAC	2964	30134-30148 (pYUXYEH2299-mcr)
	R:GTTAAATCCCAGCACC		33082-33097 (pYUXYEH2299-mcr)
pilM-IS*2*-*pilL*	F:TACCGATTTGCTCAG	1702	56744-56758 (pYUXYEH3022-mcr)
	R:GATGCTCCTCTTATTTT		58429-58445 (pYUXYEH3022-mcr)
Δ*pilU*-IS*903*-Δ*pilU*	F:AATCTCAGGCGTTGG	1271	37070-37084 (pYUXYEH3022-mcr)
	R:TACCGTGACATTTATCTTC		38322-38340 (pYUXYEH3022-mcr)
shufflon-*pilV*	F:AGGCGTAAACAAACA	1342	34996-35010 (pYUXYEH3022-mcr)
	R:ATAACCAATCGTCCC		36323-36337 (pYUXYEH3022-mcr)
groEL-IS*26*-ΔIS*Ecp1*	F:CAGGGCATCAACGAA	1492	13393-13407 (pYUXYEH933-NDM/pYUXYEH944-NDM)
	R:AGGGTGAATGTGGGTC		14869-14884 (pYUXYEH933-NDM/pYUXYEH944-NDM)
Δ*intI1*-IS*26*-ΔTn*1721*	F:TGCGTCGCCATCACA	1263	24252-24266 (pYUXYEH933-NDM/pYUXYEH944-NDM)
	R:CAGTCGTCCACGTCCAT		25498-25514 (pYUXYEH933-NDM/pYUXYEH944-NDM)

Region	sequence (5'-3')	Size (bp)	Position (bp) in plasmid
Δ*traD*-IS*Ec12*-Δ*traD*	F:AAGCCAGCGAGCAGT	2876	69068-69082 (pYUXYEH933-NDM/pYUXYEH944-NDM)
	R:CCATCTGACGACCTTCT		71927-71943 (pYUXYEH933-NDM/pYUXYEH944-NDM)
bis-IS*1294*-*dpp3*	F:GGCACGCTAAATGGT	2433	1901-1915 (pYUXYEH1271-NDM/ pYUXYEH1278-NDM)
	R:TCGGCTTTCGGTCTA		4319-4333 (pYUXYEH1271-NDM/pYUXYEH1278-NDM)
Δ*umcD*-IS*26*-Δ*cutA*	F:CAAGCGGTGTCTCCA	1364	9297-9311 (pYUXYEH1271-NDM/pYUXYEH1278-NDM)<br>7609-7623 (pYUXYEH3520-NDM/pYUXYEH3521-NDM)
	R:TTGCGGTAGGTTTCG		10646-10660 (pYUXYEH1271-NDM/pYUXYEH1278-NDM)<br>8958-8972 (pYUXYEH3520-NDM/pYUXYEH3521-NDM)
bla$_{\mathrm{NDM}}$-ΔIS*Aba125*-IS*5*-ΔIS*Aba125*	F:CGTTGGAAGCGACTG	1699	12991-13005 (pYUXYEH1271-NDM/pYUXYEH1278-NDM)<br>11303-11317 (pYUXYEH3520-NDM)
	R:TTACCTAAAGGGATTGA		14673-14689 (pYUXYEH1271-NDM/pYUXYEH1278-NDM)<br>12985-13001 (pYUXYEH3520-NDM)
Δ*taxC*-IS*1294*-Δ*taxC*	F:GATAATGGTCGTAGCC	2300	42768-42783 (pYUXYEH1271-NDM/pYUXYEH1278-NDM)
	R:GTGCCAGTAAGGAGG		45053-45067 (pYUXYEH1271-NDM/pYUXYEH1278-NDM)

hy: hypothetical protein.

Table S3 Conjugation frequencies of NDM/MCR-producing plasmids in this study

strain	plasmids	Plasmid types	Conjugation frequency
XYEH933	pYUXYEH933-NDM	F2:A-:B-	$8.32*10^{-6}$
XYEH944	pYUXYEH944-NDM	F2:A-:B-	$3.10*10^{-6}$
XYEH3314	pYUXYEH3314-NDM	F2:A-:B-	$3.82*10^{-5}$
XYEH3520	pYUXYEH3520-NDM	IncX3	$4.63*10^{-6}$
XYEH3521	pYUXYEH3521-NDM	IncX3	$2.29*10^{-5}$
XYEH3783	pYUXYEH3783-NDM	F2:A1:B1	$2.38*10^{-5}$
XYEH1135	pYUXYEH1135-mcr	IncI2	$2.48*10^{-6}$
XYEH1313	pYUXYEH1313-mcr	IncI2	$3.30*10^{-4}$
XYEH2259	pYUXYEH2259-mcr	IncI2	$8.47*10^{-4}$
XYEH2299	pYUXYEH2299-mcr	IncI2	$2.22*10^{-4}$

Table S4 Whole genomes of *Escherichia coli* strains XYEH3314, XYEH3783 and XYEH3934 in this study.

	Size (bp)	Resistance genes	Plasmid replicon
**XYEH3314**			
chromosome	4,836,682	none	
pYUXYEH3314-1	214,754	*bla*$_{TEM-1b}$/*aac(3)-IId/qnrB6/sul1/mph*(A)	IncHI2
pYUXYEH3314-NDM	93,647	*bla*$_{TEM-1b}$/*bla*$_{NDM-6}$/*aadA2*/*rmtB*/*sul1*/*dfrA12*/*mph*(A)/*erm*(B)	F2:A-:B-
pYUXYEH3314-3	30,965	*bla*$_{TEM-1b}$	-
pYUXYEH3314-4	21,237	*bla*$_{CTX-M-55}$/*strAB/aac(6')-Ib-cr/tet(A)/floR/sul2/dfrA27/arr-3*	IncQ1
pYUXYEH3314-5	4,064	none	
pYUXYEH3314-6	3,174	none	
pYUXYEH3314-7	2,461	none	
**XYEH3783**			
chromosome	4,982,136	none	
pYUXYEH3783-NDM	168,068	*bla*$_{TEM-1b}$/*bla*$_{NDM-5}$/*aac(3)-IId/aadA1/aadA2/aadA22/tet*(A)/*cmlA1* /*floR/qnrS1/sul3/dfrA12*	F2:A1:B1
pYUXYEH3783-2	33,750	none	-
**XYEH3934**			
chromosome	4,709,641	*strAB*	
pYUXYEH3934-1	188,533	*bla*$_{CTX-M-14}$/*aadA1/aadA2/tet*(A)/*cmlA1/qnrS1/fosA3/sul3/dfrA12/* *dfrA14/mef(B)*	IncFIB(K)
pYUXYEH3934-2	31,816	*aadA2/tet*(A)/*tet*(X4)/*floR*/*lnu*(F)	IncX1
pYUXYEH3934-3	6,756	none	-
pYUXYEH3934-4	2,445	none	-

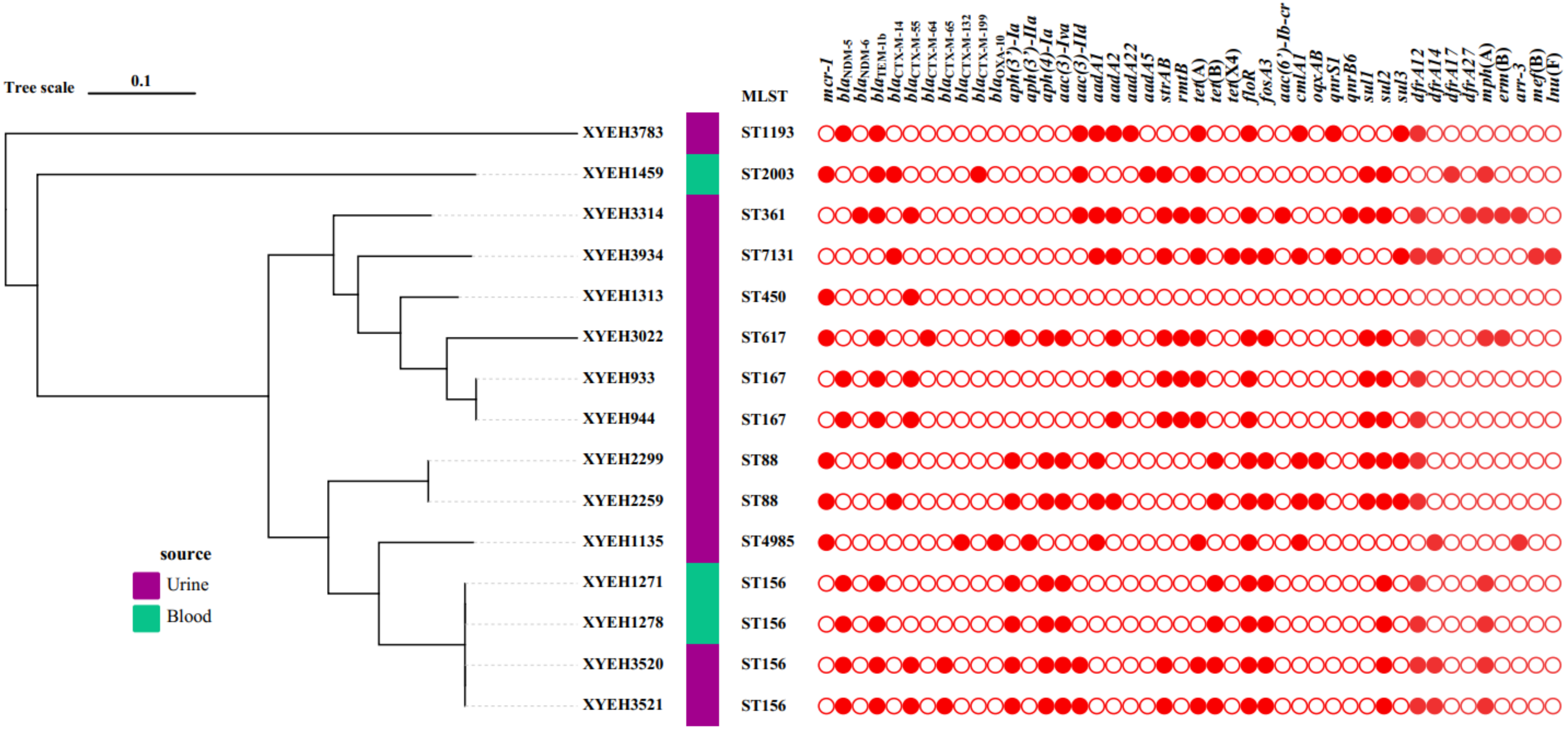

Figure S1 The maximum likelihood tree of *bla*$_{NDM}$/*mcr-1*/*tet*(X4)-positive *E. coli* isolates in this study. Antibiotic resistance genes are present as red solid circle.

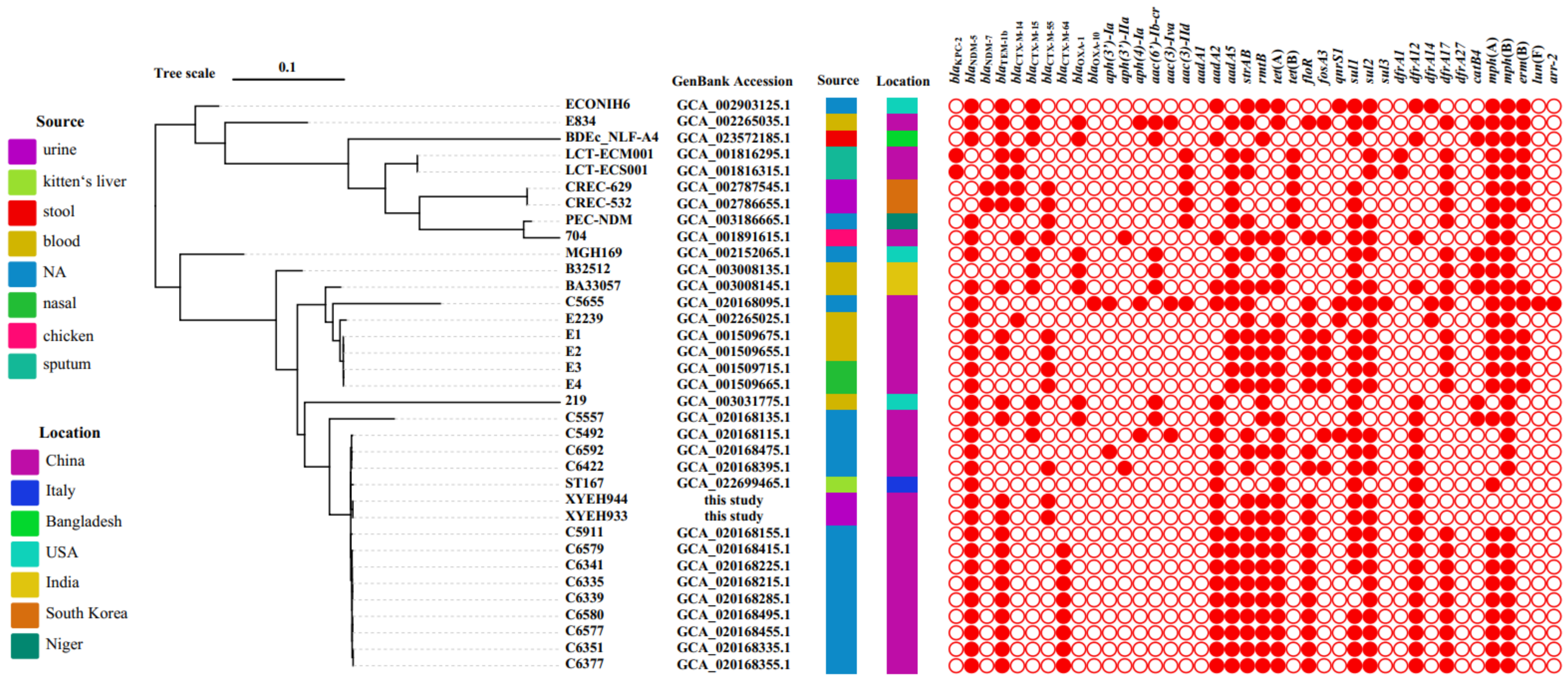

Figure S2 The maximum likelihood tree of *bla*$_{NDM-5}$-positive ST167 *E. coli* isolates in this study compared with *E. coli* ST167 isolates from NCBI Assembly database based on cgSNP analysis. The presence of resistance genes is indicated as red solid circle.
